# Prognostic value of preoperative low bone mineral density in patients with digestive cancers: a systematic review and meta-analysis

**DOI:** 10.1007/s11657-022-01060-6

**Published:** 2022-02-11

**Authors:** Jun Watanabe, Akihiro Saitsu, Atsushi Miki, Kazuhiko Kotani, Naohiro Sata

**Affiliations:** 1 Department of Surgery, Division of Gastroenterological, General and Transplant Surgery, Jichi Medical University, Shimotsuke-City, Tochigi, 329-0498 Japan; 2 Division of Community and Family Medicine, Jichi Medical University, Shimotsuke-City, Tochigi, Japan

**Keywords:** Bone mineral density, Digestive system neoplasms, Meta-analysis, Mortality, Osteopenia, Prognosis, Recurrence, Systematic review

## Abstract

**Purpose:**

Osteopenia typically presents low bone mineral density (BMD) and has recently been reported as a prognostic factor in various cancers. However, the prognostic value of osteopenia in digestive tract cancers remains to be defined. We aimed to review the prognostic value of preoperative osteopenia in patients with digestive cancers.

**Methods:**

Cohort studies evaluating the prognostic value of preoperative osteopenia in digestive cancers (colorectal, esophageal, hepatic, bile duct, and pancreatic cancer) were searched using electronic databases and trial registries. The exposure was defined as low BMD estimated by computed tomography at 11^th^ thoracic vertebra, while comparator was normal BMD. The primary outcomes were overall survival and recurrence-free survival for osteopenia. Random effect meta-analyses were performed. The Grading of Recommendations, Assessment, Development, and Evaluation approach was used to assess the certainty of evidence.

**Results:**

A total of 11 studies (2230 patients) were included. Osteopenia was an independent risk factor for overall survival (hazard ratio [HR] = 2.02, 95% CI = 1.47 to 2.78; *I*^2^ = 74%), along with sarcopenia. Osteopenia also predicted poor recurrence-free survival (HR = 1.96, 95% CI = 1.36 to 2.81; *I*^2^ = 85%). In subgroup analyses, osteopenia predicted prognosis in colorectal, esophageal, hepatic, and bile duct cancers, but not in pancreatic cancer. The certainty of the evidence was low due to inconsistency and publication bias.

**Conclusion:**

Osteopenia may be independently associated with poor prognosis in patients with digestive tract cancer. Further studies are needed to establish the relevance of osteopenia in the operative prognosis of these patients.

**Supplementary Information:**

The online version contains supplementary material available at 10.1007/s11657-022-01060-6.

## Introduction

Digestive tract organs, including esophageal, gastric, colon, pancreatic, and hepatic organs, can develop cancers; digestive tract cancer is a leading cause of cancer-related mortality worldwide [1]. The prognosis of most of digestive tract cancers is unfavorable and unsatisfactory [2]. Tumor factors such as tumor size, number, and metastasis are important determinants of prognosis for patients with digestive tract cancers, but patient-level factors can have a significant impact on prognosis [3]. As a patient-level factor, sarcopenia has been shown to be a poor prognostic factor in patients who underwent surgery for digestive tract cancers, such as esophageal, gastric, colon, pancreatic, and hepatic cancers [4, 5].

Osteopenia, a condition of low bone mineral density (BMD), is another important patient-level factor in cancer treatment because low BMD is associated with a higher risk of falls, fractures, institutionalization, and death, negatively impacting health-related quality of life, and ultimately, the prognosis [6]. A previous study demonstrated that low BMD, a surrogate marker for osteopenia, was an independent risk factor for poor prognosis that precedes sarcopenia [7]. Computed tomography (CT)-derived BMD assessment correlates with dual-energy X-ray absorptiometry (DXA), the gold standard for osteoporosis diagnosis [8], with BMD being analyzed by measuring the mean pixel density of the thoracic vertebral trabeculae on preoperative CT [9]. Although the prognostic value of preoperative osteopenia in patients with digestive tract cancers has been reported [7, 10–13], no systematic review of osteopenia has been performed in these patients.

We hypothesized that preoperative osteopenia (indicative of low BMD) could have a negative impact on long-term outcomes in patients who underwent surgery for digestive tract cancers. Thus, the aim of this study was to review the prognostic value of preoperative osteopenia in patients who underwent resection for digestive tract cancers. Our study also included sarcopenia, knowing that the relevance of osteopenia on prognosis was independent of sarcopenia. This is because sarcopenia (which is a pathology of decreased muscles) can coexists in osteopenic conditions as named osteosarcopenia [10].

## Methods

### Protocol and registration

The results of the present review are presented according to the Preferred reporting Items for Systematic Review and Meta-analysis (PRISMA) 2020 [11]. This protocol was registered in PROSPERO (https://www.crd.york.ac.uk/prospero/).

### Inclusion criteria of the articles for the review

Studies were included using the PECO framework: participant, exposure, comparator, and outcome. The participants were patients who underwent surgery for digestive tract cancer (esophageal, gastric, colorectal, liver, pancreatic, biliary tract, and gallbladder cancer). Each digestive tract cancer (esophageal, gastric, colorectal, liver, pancreatic, biliary tract, and gallbladder cancer) was pathologically diagnosed. The exposure was the presence of preoperative osteopenia defined as preoperative BMD calculated as the average pixel density (HU) within a circle in the midvertebral core at the bottom of the 11^th^ thoracic vertebra on preoperative computed tomography. This assessment correlates with DXA, the gold standard for osteoporosis diagnosis [8, 9]. The cutoff values for BMD were adopted via comparison with those of healthy adults or DXA values, and the original authors’ own cutoff values were also accepted. The comparators were preoperative normal BMD. The primary outcomes were overall survival (OS) and recurrence-free survival (RFS) for osteopenia. The secondary outcome was the OS for sarcopenia. Sarcopenia was diagnosed according to body composition (using computed tomography, DXA, and bioelectrical impedance) or by the European Working Group on Sarcopenia in Older People 2 guidelines [4]. No language, country, observation period, or publication year restrictions were applied. Reviews and case series or reports were excluded.

### Search method

The electronic databases of MEDLINE (PubMed), the Cochrane Central Register of Controlled Trials (Cochrane Library), and EMBASE (Dialog) (Appendix 1), and trial registries in the World Health Organization International Clinical Trials Platform Search Portal (ICTRP), and ClinicalTrials.gov (Appendix 2) were searched until August 9, 2021. The reference lists of studies, including international guidelines [12] as well as the reference lists of eligible studies, and articles citing eligible studies were checked. The original authors were asked for unpublished or additional data.

### Data collection and analysis

Two reviewers (JW and AS) independently screened the studies based on the inclusion and exclusion criteria and extracted data from the eligible studies. Any disagreements between the two reviewers were resolved by discussion, with disagreements being resolved by a third reviewer acting as an arbiter (KK). Two reviewers (JW and AS) independently evaluated the risk of bias using the Quality In Prognosis Studies tool [13, 14].

Random-effects meta-analyses were performed using Review Manager software (RevMan 5.4.2). Hazard ratios (HRs) and 95% confidence intervals (CIs) for OS and RFS were pooled. Statistical heterogeneity was evaluated using visual inspection of the forest plots and calculating the *I*^2^ statistic (*I*^2^ values of 0 to 40%: might not be important; 30 to 60%: may represent moderate heterogeneity; 50 to 90%: may represent substantial heterogeneity; 75 to 100%: considerable heterogeneity) [15]. Publication bias was investigated by searching for unpublished trials using the clinical trial registry system (ClinicalTrials.gov and ICTRP). To assess the outcome reporting bias, we compared the outcomes defined in the trial protocols with the outcomes reported in the publications. Potential publication bias was assessed by visual inspection of the funnel plot and Egger’s test according to the Cochrane handbook [15]. Subgroup analyses were performed for each cancer type. Sensitivity analysis planned in our protocol could not be performed because of the lack of studies using imputed statistics.

Two reviewers (JW and AS) evaluated the certainty of evidence using the five Grading of Recommendations Assessment, Development, and Evaluation (GRADE) considerations of within- and across-study risk of bias (limitations in the study design and execution or methodological quality), inconsistency (or heterogeneity), indirectness of evidence, and imprecision of the effect estimates and risk of publication bias [16]. Disagreements between the two reviewers were discussed and resolved with a third reviewer (KK) acting as an arbiter, if necessary. A summary of findings table was made for the outcomes based on the Cochrane handbook [15].

## Results

**Fig. 1:**
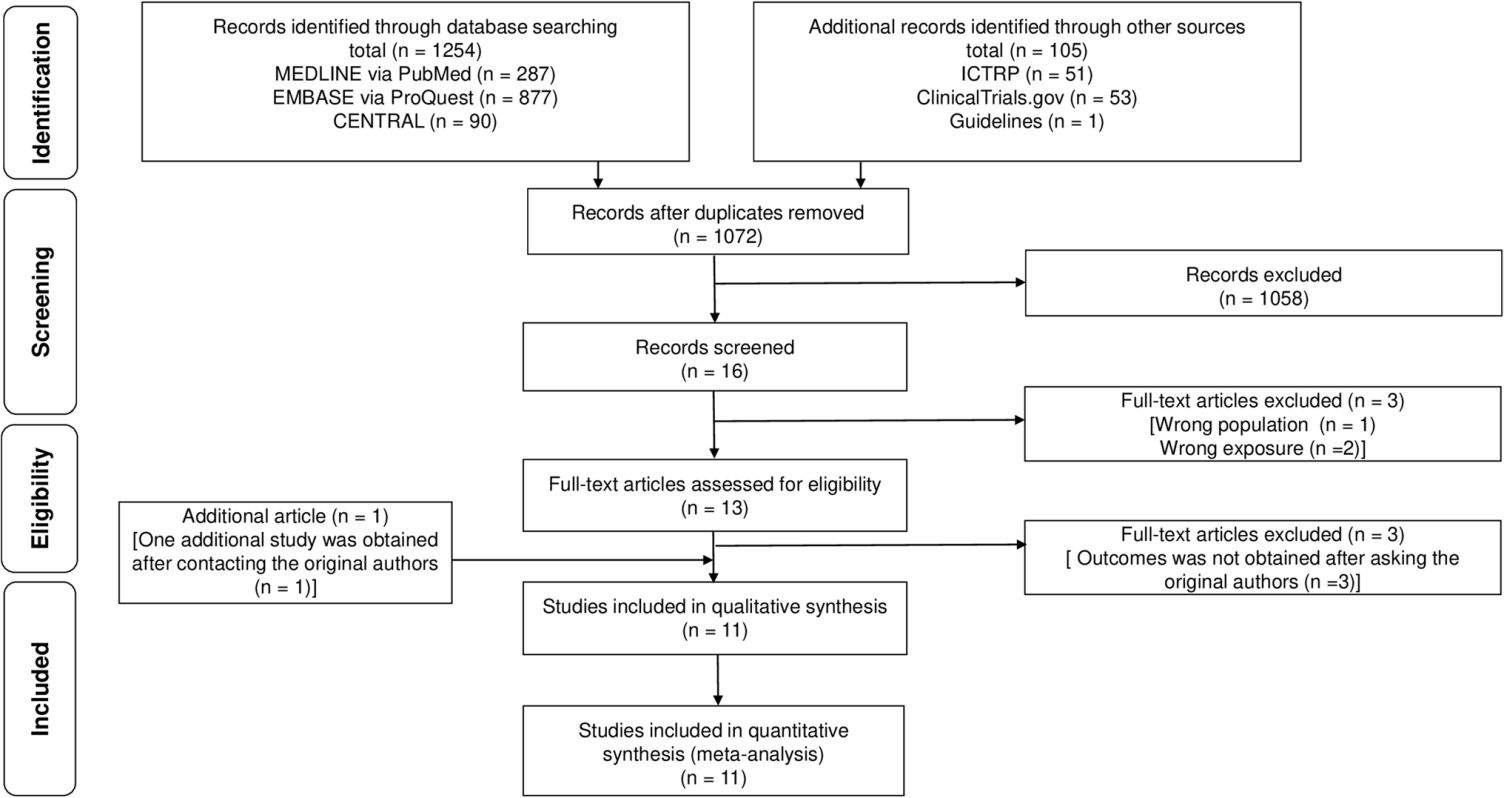
Flow of the study selection process

Figure 1 illustrates the process of the study selection. After screening 1072 records, we retained 16 studies for full-text reading. Three studies were excluded because one study had an incorrect population [17], and the other two were incorrect exposures [18, 19]. Three additional studies were excluded because missing data could not be obtained after asking the original authors [20–22]. After contacting the original authors, we included an additional study. Finally, 11 studies (2230 patients) were included for the meta-analysis [23–33].

Table 1 shows the characteristics of the included studies [23–33]. Of the 11 studies, three studies focused on pancreatic cancer [25, 26, 28], two on colorectal liver metastases [29, 30], two on extrahepatic cholangiocarcinoma [24, 32], one on esophageal cancer [31], one on colon cancer [33], and two on hepatocellular carcinoma [23, 27]. Six studies adopted cutoff values for BMD from previous studies [23, 25, 33, 27–29], while the other five studies adopted their own cutoff values [24, 26, 30–32]. There was no statistically significant difference in the association between low BMD and normal BMD in five studies where patients received neoadjuvant chemotherapy [25, 26, 29–31]. The risk of bias for study participation, study attrition, outcome measurements, and statistical analysis were low. The risk of bias for prognostic factor measurement was moderate in five studies as they had their own BMD cutoff values [24, 26, 30–32].

**Table 1:** The characteristics of the included studies

Authors [ref no.]	Year	Cancer type	Subject no.	Age	Osteopenia no. (%)	Cutoff values of BMD (HU) (male/female)	Neoadjuvant chemotherapy no. (%)	Sarcopenia	QUIPS
Miyachi [23]	2019	HCC	465	69	273 (59)	160	NR	PMI	Low
Yao [24]	2019	ECC	181	68	124 (69)	169	NR	PMI	Moderate
Motomura [25]	2020	PC	91	75	34 (37)	308.82 − 2.49*age / 311.84 − 2.41*age	4/91 (4)	SMI	Low
Sharshar [26]	2020	PC	275	71	136 (50)	137.5/128.8	32/275 (11)	PMI	Moderate
Toshima [27]	2020	HCC	193	58	103 (53)	308.82 − 2.49*age / 311.84 − 2.41*age	NR	SMA	Low
Abe [28]	2021	PC	56	72	27 (48)	160	0/56 (0)	SMI	Low
Furukawa [29]	2021	CRLM	118	68	66 (56)	308.82 − 2.49*age / 311.84 − 2.41*age	41/118 (35)	PMA	Low
Ikuta [30]	2021	CRLM	281	66	143 (51)	141	168/281 (60)	NR	Moderate
Takahashi [31]	2021	EC	229	68	70 (31)	122.23	127/229 (55)	SMI	Moderate
Tamura [32]	2021	ECC	111	72	8 (7)	75/74	NR	SMI	Moderate
Kamada [33]	2021	CC	230	67	43 (19)	308.82 − 2.49*age / 311.84 − 2.41*age	NR	SMI	Low

*BMD* bone mineral density, *CC* colon cancer, *CRLM* colorectal liver metastases, *ECC* extrahepatic cholangiocarcinoma, *HCC* hepatocellular carcinoma liver transplant donor, *NR* not reported, *PC* pancreatic cancer, *PMI* psoas muscle index, *QUIPS* the Quality In Prognosis Studies, *SMA* skeletal muscle area, *SMI* skeletal muscle index

### Prognostic value of preoperative low BMD in patients with digestive tract cancers

Table 2 summarizes the findings using the GRADE approach. The certainty of the evidence was low to moderate due to inconsistency and publication bias.

**Table 2:** Summary of findings

Prognostic value of preoperative osteopenia in patients with digestive cancers				
Patients: patients with digestive cancers, Exposure: Osteopenia, Comparison: Non-osteopenia				
Outcomes	Relative effect (95% CI)	Patient number (studies)	Certainty of the evidence (GRADE)	Comments
Overall survival for osteopenia	HR 2.02 (1.47 to 2.78)	2230 (11 studies)	Low ^a,b^	Osteopenia may reduce overall survival.
Recurrent-free survival for osteopenia	HR 1.96 (1.36 to 2.81)	2037 (10 studies)	Low ^a,b^	Osteopenia may reduce recurrent-free survival.
Overall survival for sarcopenia	HR 1.73 (1.26 to 2.38)	1665 (8 studies)	Moderate ^a^	Sarcopenia likely reduce overall survival.

GRADE Working Group grades of evidence; High certainty: We are very confident that the true effect lies close to that of the estimated effect. Moderate certainty: We are moderately confident in the estimated effect. The true effect is likely to be close to the estimated effect, but there is a possibility that it is substantially different. Low certainty: Our confidence in the estimated effect is limited: The true effect may be substantially different from the estimated effect. Very low certainty: We have very little confidence in the estimated effect. The true effect is likely to be substantially different from the estimated effect

Abbreviations: *CI* confidence interval, *HR* hazard ratio

^a^Downgraded one point as inconsistency due to substantial heterogeneity

^b^Downgraded one point as publication bias due to high publication bias

#### OS for low BMD

**Fig. 2:**
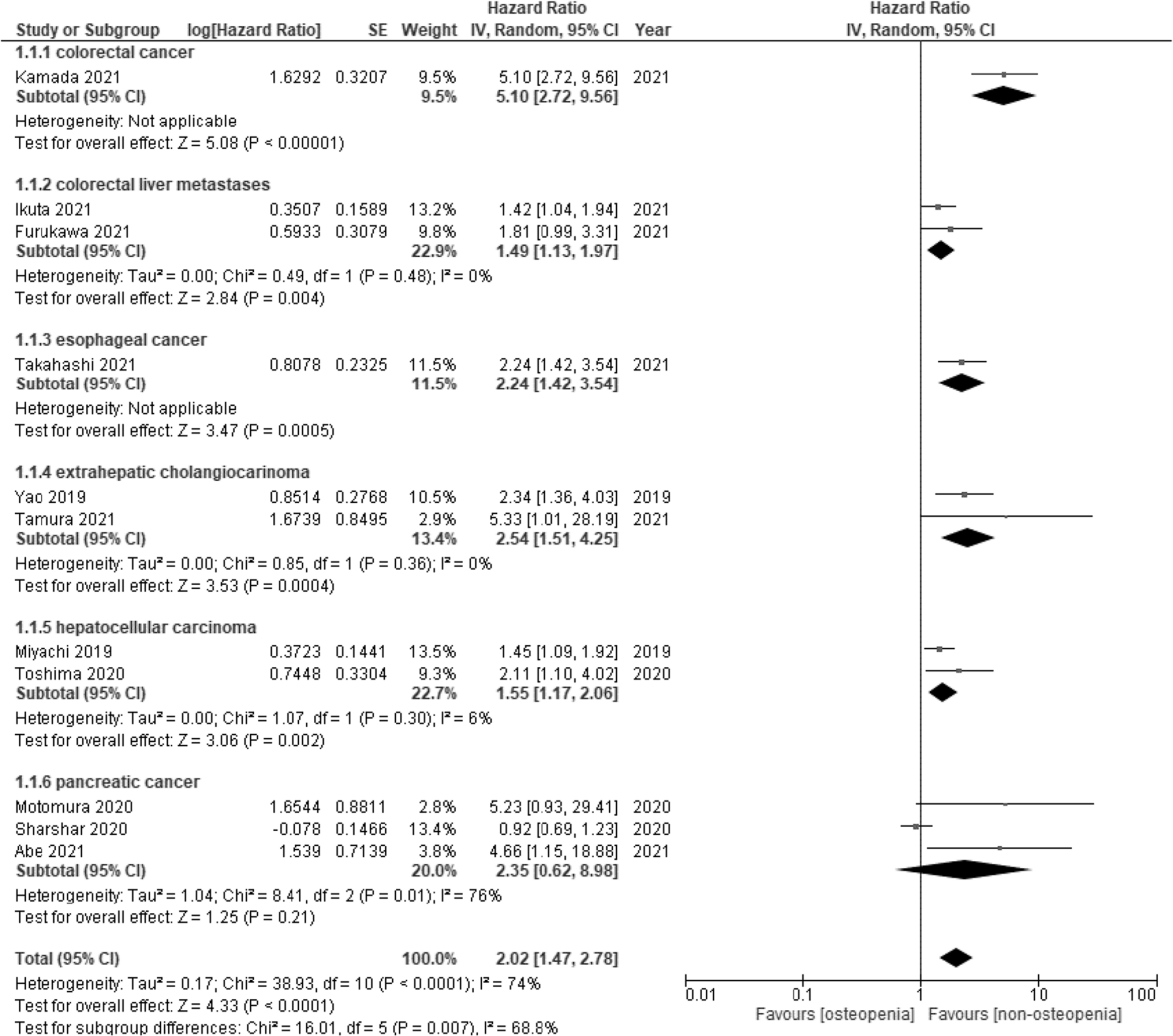
Forest plot of overall survival for osteopenia

OS for low BMD was measured in 11 studies. Low BMD was a significant risk factor for OS (HR = 2.02, 95% CI = 1.47 to 2.78; I^2^ = 74%) (Fig. 2). Subgroup analysis of cancer types showed significant differences (*p* = 0.007). Low BMD in colorectal cancer (HR = 5.10, 95% CI = 2.72 to 9.56), colorectal liver metastases (HR = 1.49, 95% CI = 1.13 to 1.97; *I*^2^ = 0%), esophageal cancer (HR = 2.24, 95% CI = 1.42 to 3.54), extrahepatic cholangiocarcinoma (HR = 2.54, 95% CI = 1.51 to 4.25; *I*^2^ = 0%), and hepatocellular carcinoma (HR = 1.55, 95% CI = 1.17 to 2.06; *I*^2^ = 6%) were a significant risk factor for OS, while low BMD in pancreatic cancer (HR = 2.35, 95% CI = 0.62 to 8.98; *I*^2^ = 76%) was not significant.

#### RFS for low BMD

**Fig. 3:**
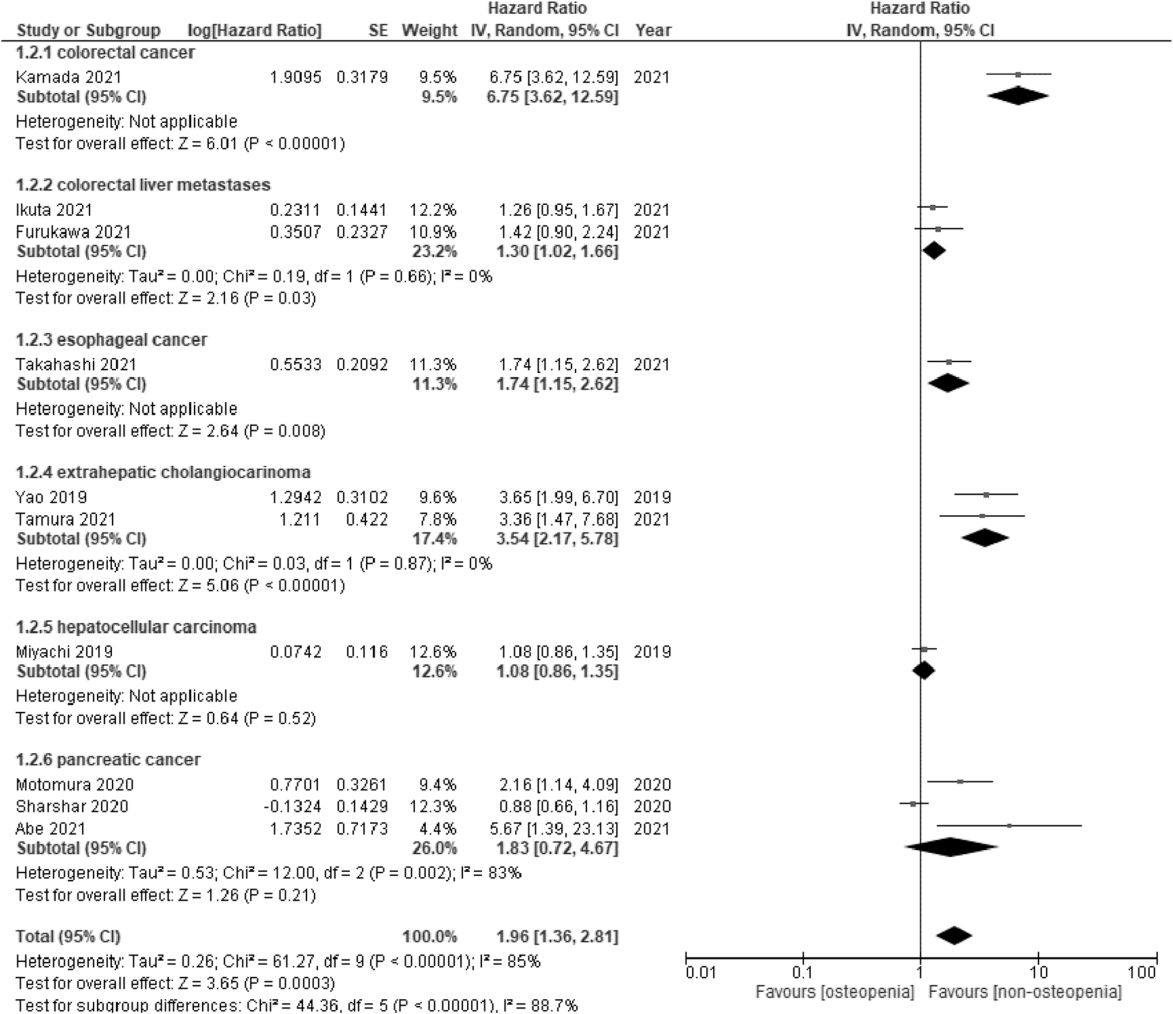
Forest plot of recurrence-free survival for osteopenia

The RFS for low BMD was measured in ten studies. Low BMD was a significant risk factor for RFS (HR = 1.96, 95% CI = 1.36 to 2.81; *I*^2^ = 85%) (Fig. 3). Subgroup analysis of cancer types showed a significant difference (*p* < 0.00001). Low BMD in colon cancer (HR = 6.75, 95% CI = 3.62 to 12.56), colorectal liver metastases (HR = 1.30, 95% CI = 1.02 to 1.66; *I*^2^ = 0%), esophageal cancer (HR = 1.74, 95% CI = 1.15 to 2.62), and extrahepatic cholangiocarcinoma (HR = 3.54, 95% CI = 2.17 to 5.78; *I*^2^ = 0%) were significant risk factors for RFS, while low BMD in hepatocellular carcinoma (HR = 1.08, 95% CI = 0.86 to 1.35) and pancreatic cancer (HR = 1.83, 95% CI = 0.72 to 4.67; *I*^2^ = 83%) were not significant.

### OS for sarcopenia

**Fig. 4:**
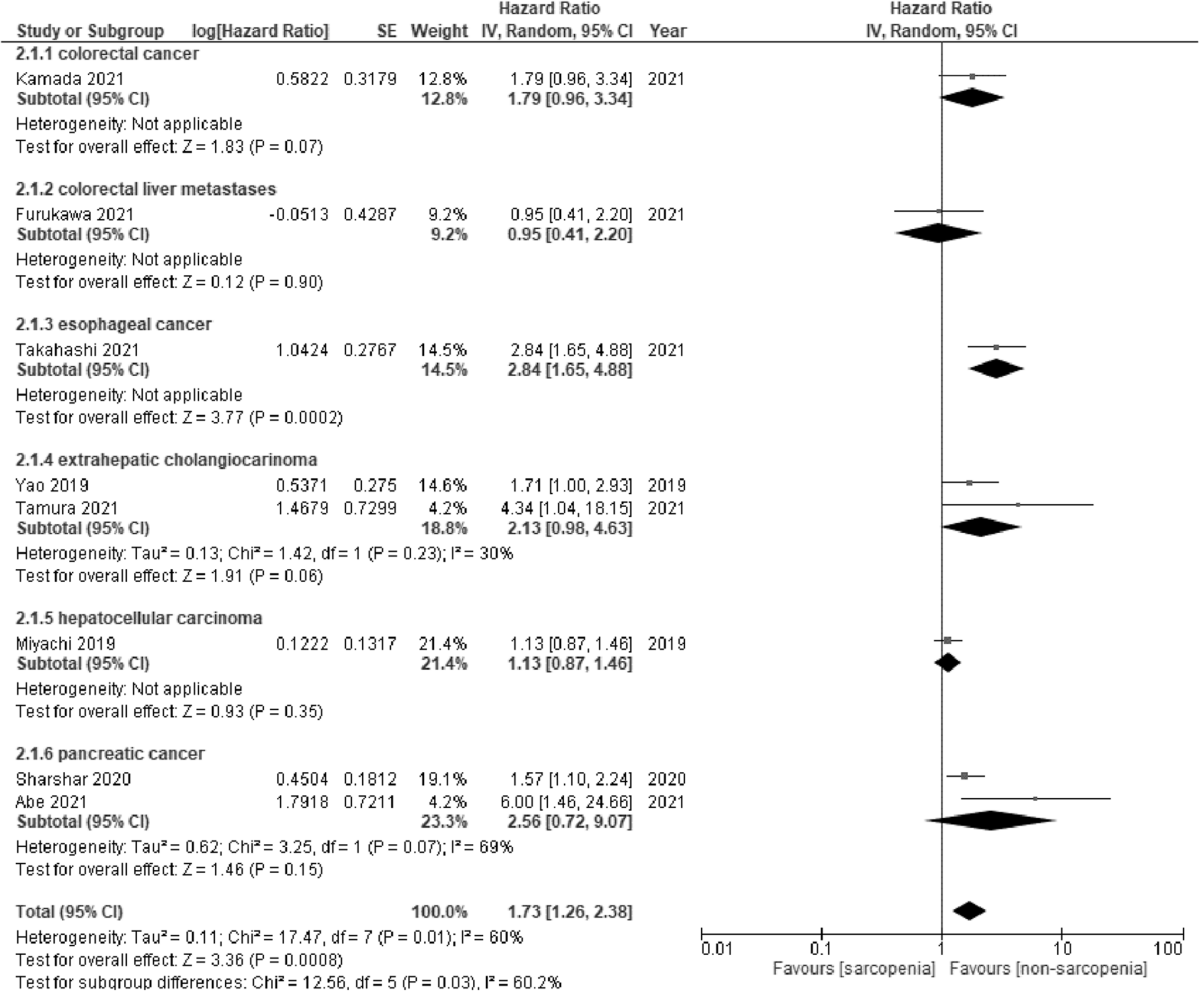
Forest plot of overall survival for sarcopenia

The OS for sarcopenia was measured in eight studies. Sarcopenia was a significant risk factor for OS (HR = 1.73, 95% CI = 1.26 to 2.38; *I*^2^ = 60%) (Fig. 4). Subgroup analysis of cancer types showed significant differences (*p* = 0.03). Sarcopenia in esophageal cancer (HR = 2.84, 95% CI = 1.65 to 4.88) was a significant risk factor for OS, while sarcopenia in colon cancer (HR = 1.79, 95% CI = 0.96 to 3.34), colorectal liver metastases (HR = 0.95, 95% CI = 0.41 to 2.20), extrahepatic cholangiocarcinoma (HR = 2.13, 95% CI = 0.98 to 4.63; *I*^2^ = 30%), hepatocellular carcinoma (HR = 1.13, 95% CI = 0.87 to 1.46), and pancreatic cancer (HR = 2.56, 95% CI = 0.72 to 9.07; *I*^2^ = 69%) was not significant.

### Publication bias

**Fig. 5:**
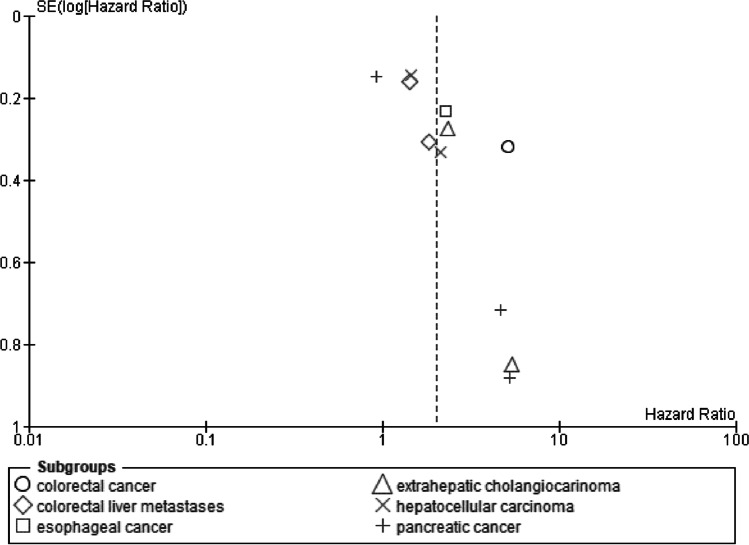
Funnel plot of overall survival for osteopenia

**Fig. 6:**
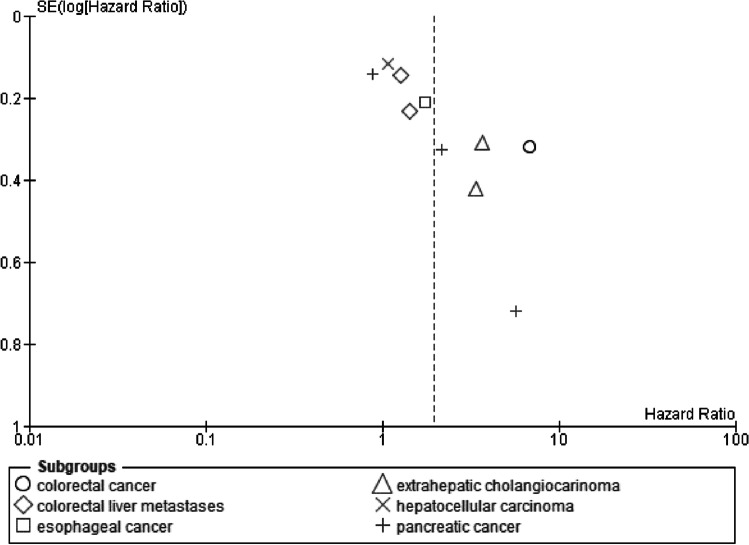
Funnel plot of recurrence-free survival for osteopenia

Regarding the publication bias of OS and RFS for low BMD, the funnel plots were asymmetric, and the lower left parts were missing, suggesting a potential publication bias (Egger test, *p* = 0.0083 and 0.0028, respectively) (Figs. 5 and 6). Regarding the publication bias of OS for sarcopenia, the funnel plot and Egger test could not be performed because less than 10 trials were found for each outcome according to the Cochrane handbook [15].

## Discussion

Our study demonstrated that low BMD was an independent risk factor for OS and RFS. Sarcopenia was also an independent risk factor for OS. Our first systematic review and meta-analysis on the prognostic value of preoperative osteopenia (indicative of low BMD) may imply the importance of preoperative assessment of osteopenia in patients who underwent resection of digestive tract cancers.

In contrast to the potential implication, the mechanism of the effect of osteopenia on poor prognosis remains to be elucidated. One possible reason for this is that cachexia could stimulate osteoclast, which causes osteopenia [34]. Osteoclasts are formed and activated by cytokines derived from cancer cells, such as PTHrP, interleukin (IL)-1, IL-6, and IL-8, which activate the RANK/RANKL (receptor activator of NF-κB ligand) signaling mechanism [35]. NF-κB is also a possible cause of sarcopenia [34] and a key molecule in the progression of digestive tract cancers [36–38]. Therefore, these findings suggest that osteopenia may be associated with sarcopenia and poor prognosis in digestive tract cancers.

In a previous study, patients who received neoadjuvant chemoradiotherapy tended to have reduced BMD levels [19]. However, in the studies included in this review, preoperative radiation therapy was not performed, and neoadjuvant chemotherapy did not affect prognosis [25, 26, 29, 30]. This review found the relationship between osteopenia and prognosis to be comparably high (i.e., HR > 2); however, the reasons for this relationship remain unclear. As there may be hidden factors apart from chemotherapy and/or radiotherapy, this relationship merits further investigation.

To date, we have found one systematic review showing the significance of low BMD on breast cancer among various cancers [36]. In breast cancer, we consider the influence of menopause and hormone replacement therapy, which affects in vivo estrogen levels, on prognosis; this is to some degree associated with BMD [37, 38]. As digestive cancers are not affected by estrogen, the influence of low BMD on prognosis was directly evaluated in this review.

The poor prognosis of osteopenia in patients with digestive tract cancers is debatable in relation to sarcopenia. In a previous study, osteopenia was an independent risk factor for poor prognosis that precedes sarcopenia [7]. In our review, osteopenia was associated with lower OS compared to sarcopenia in all cancers except pancreatic cancer. Differences in cutoff values of osteopenia, sex, and age may be a reason why osteopenia was not identified as a risk factor for OS in pancreatic cancer [39]. However, the subgroup analysis could not examine the results separately for each sex or age because the number of studies for each cancer included in this review was small. Our results indicate that osteopenia may be a poor prognostic factor that precedes sarcopenia; however, further studies are warranted to investigate in detail the relationship between osteopenia and sarcopenia on the prognostic values in patients with digestive tract cancers.

Methods to improve osteopenic conditions include exercise and oral nutritional support [40]. Exercise, especially progressive resistance training, weight-bearing impact activities or multimodal programs, is an important approach to manage cancer-related bone loss [41, 42]. Preoperative nutritional support, especially calcium intake through diet, supplements, or both and vitamin D supplementation, may prevent osteopenia because vitamin D deficiency is a significant risk factor for bone density loss [18]. However, the level of evidence supporting these recommendations is limited.

A potential publication bias was detected in OS for osteopenia. However, in accordance with the PRISMA statement [11], protocols were searched using clinical trial registry systems (ClinicalTrials.gov and ICTRP) to examine unpublished trials, which were not identified. Furthermore, we contacted the original authors and searched for potential unpublished studies whenever possible.

This study has several limitations. First, the number of studies for each cancer included in this review was small. However, a rigorous methodology was adopted, including a comprehensive search, according to the PRISMA statement. Second, DXA, the gold standard for assessing osteopenia, was not available, and the cutoff values for osteopenia differed between studies. DXA scans were not available at all centers, and additional costs were borne by patients, while preoperative CT was available for all preoperative patients with digestive tract cancers and is a simple way to measure BMD. Third, the results of the Japanese studies included in this review may not be applicable to patients with digestive tract cancers in Western countries. Further international multicenter studies are needed to assess the impact of osteopenia on prognosis, because these limitations make the supporting evidence less certain, and drawing valid conclusions is also made more difficult.

## Conclusion

The findings of the present systematic review and meta-analysis indicated that preoperative osteopenia (indicative of low BMD) in patients with digestive tract cancers likely predicted poor prognosis. Considering that osteopenia affected prognosis independently of sarcopenia in each cancer, osteopenia may be an early marker of poor prognostic factors that precede sarcopenia. Further studies are needed to clarify the prognostic impact of osteopenia in patients with digestive tract cancers.

## Supplementary Information

Appendix 1. The electronic database search strategy (PNG 3982 kb)

High resolution image (TIFF 15629 kb)

Appendix 2. The trial registry search strategy (PNG 3896 kb)

High resolution image (TIFF 15629 kb)

ESM 3 (DOCX 16 kb)

## Abbreviations

BMD: Bone mineral density
CI: Confidence interval
CT: Computed tomography
DXA: Dual-energy x-ray absorptiometry
GRADE: Grading of Recommendations Assessment, Development, and Evaluation
HR: Hazard ratio
OS: Overall survival
PRISMA: The Preferred Reporting Items for Systematic Review and Meta-Analysis
RFS: Recurrence-free survival
