# Supplementary material for: Prognostic value of preoperative low bone mineral density in patients with digestive cancers: a systematic review and meta-analysis
Source: Arch Osteoporos. 2022 Feb 11;17(1):33. doi: 10.1007/s11657-022-01060-6 (PMC8837550; doi:10.1007/s11657-022-01060-6)
Supplement: Supplementary file 5 — (DOCX 16 kb) [file 11657_2022_1060_MOESM3_ESM.docx]

Appendix 1: The electronic database search strategy

MEDLINE (PubMed) search strategy

#1. "bone mineral density"[tiab]

#2. "Digestive System Neoplasms"[Mesh]

#3. gastrointestin*[tiab] OR gastro‐intestin*[tiab] OR intestin*[tiab] OR bowel*[tiab] OR colon*[tiab] OR colorectal[tiab] OR rectal*[tiab] OR stomach*[tiab] OR gastric*[tiab] OR esophageal*[tiab] OR bil*[tiab] OR cholangio*[tiab] OR pancreat*[tiab] OR hepato*[tiab] OR liver*[tiab]

#4. "Neoplasms"[Mesh]

#5. (neoplasm*[tiab] OR cancer*[tiab] OR carcinoma*[tiab] OR tumor*[tiab] OR tumour*[tiab])

#6. #4 OR #5

#7. #3 AND #6

#8. #2 OR #7

#9. #1 AND #8

("bone mineral density"[tiab]) AND (((gastrointestin*[tiab] OR gastro‐intestin*[tiab] OR intestin*[tiab] OR bowel*[tiab] OR colon*[tiab] OR colorectal[tiab] OR rectal*[tiab] OR stomach*[tiab] OR gastric*[tiab] OR esophageal*[tiab] OR bil*[tiab] OR cholangio*[tiab] OR pancreat*[tiab] OR hepato*[tiab] OR liver*[tiab])) AND (("Neoplasms"[Mesh]) OR ((neoplasm*[tiab] OR cancer*[tiab] OR carcinoma*[tiab] OR tumor*[tiab] OR tumour*[tiab]))))

EMBASE (Dialog) search strategy

S1 ab(bone mineral density) OR ti(bone mineral density)

S2 EMB.EXACT.EXPLODE("digestive system tumor")

S3 (ab(gastrointestin*) OR ti(gastrointestin*) OR ab(gastro‐intestin*) OR ti(gastro‐intestin*) OR ab(intestin*) OR ti(intestin*) OR ab(gastro‐intestin*) OR ti(gastro‐intestin*) OR ab(bowel*) OR ti(bowel*) OR ab(colon*) OR ti(colon*) OR ab(colorectal) OR ti(colorectal) OR ab(rectal*) OR ti(rectal*) OR ab(stomach*) OR ti(stomach*) OR ab(gastric*) OR ti(gastric*) OR ab(esophageal*) OR ti(esophageal*) OR ab(bil*) OR ti(bil*) OR ab(cholangio*) OR ti(cholangio*) OR ab(pancreat*) OR ti(pancreat*) OR ab(hepato*) OR ti(hepato*) OR ab(liver*) OR ti(liver*))

S4 EMB.EXACT.EXPLODE("neoplasm")

S5 ab(neoplasm*) OR ti(neoplasm*) OR ab(cancer*) OR ti(cancer*) OR ab(carcinoma*) OR ti(carcinoma*) OR ab(tumor*) OR ti(tumor*) OR ab(tumour*) OR ti(tumour*)

S6 S4 OR S5

S7 S3 AND S6

S8 S2 OR S7

S9 S1 AND S8

CENTRAL (Cochrane Library) search strategy

#1. "bone mineral density":ti,ab

#2. MeSH descriptor: [Digestive System Neoplasms] explode all trees

#3. (gastrointestin* OR gastro‐intestin* OR intestin* OR bowel* OR colon* OR colorectal OR rectal* OR stomach* OR gastric* OR esophageal* OR bil* OR cholangio* OR pancreat* OR hepato* OR liver*):ti,ab

#4. MeSH descriptor: [Neoplasms] explode all trees

#5. (neoplasm* OR cancer* OR carcinoma* OR tumor* OR tumour*):ti,ab

#6. #4 OR #5

#7. #3 AND #6

#8. #2 OR #7

#9. #1 AND #8

Appendix 2: The trial registry search strategy

ICTRP search strategy

Bone mineral density AND cancer

ClinicalTrials.gov search strategy

Condition or disease: (gastrointestin* OR gastro‐intestin* OR intestin* OR bowel* OR colon* OR colorectal OR rectal* OR stomach* OR gastric* OR esophageal* OR bil* OR cholangio* OR pancreat* OR hepato* OR liver*) AND cancer

Intervention: bone mineral density
